# Supplementary material for: Active site specificity profiling datasets of matrix metalloproteinases (MMPs) 1, 2, 3, 7, 8, 9, 12, 13 and 14
Source: Data Brief. 2016 Feb 22;7:299–310. doi: 10.1016/j.dib.2016.02.036 (PMC4777984; doi:10.1016/j.dib.2016.02.036)
Supplement: Supplementary file 10 — Supplementary material [file mmc10.zip › WebPICS_hMMP3_T_1%/P3prime.html]

 

PICS results


|  |  |
| --- | --- |
| **P3prime\_A**  25 in 199 sites   12.6 %    effects > 10 perc. pnts.   (vice-versa in brackets)  P3\_I: 15.5 (22.7)   P1\_A: 12.4 (13.5) |  |
| **P3prime\_C**  3 in 199 sites   1.5 %    effects > 10 perc. pnts.   (vice-versa in brackets)  P2\_Q: 59.2 (11.8)   P1\_S: 58.7 (11.0) |  |
| **P3prime\_E**  14 in 199 sites   7.0 %    effects > 10 perc. pnts.   (vice-versa in brackets)  P3\_I: 12.9 (10.6)   P1\_S: 20.6 (18.0) |  |
| **P3prime\_G**  30 in 199 sites   15.1 %    effects > 10 perc. pnts.   (vice-versa in brackets)  P2\_M: 13.2 (56.3)   P1prime\_V: 11.1 (10.7) |  |
| **P3prime\_H**  6 in 199 sites   3.0 %    effects > 10 perc. pnts.   (vice-versa in brackets)  P2\_F: 28.8 (19.2)   P1\_P: 57.7 (19.2)   P1prime\_Q: 27.8 (15.2)   P1prime\_W: 13.7 (13.7)   P2prime\_Q: 27.8 (15.2) |  |
| **P3prime\_Q**  12 in 199 sites   6.0 %    effects > 10 perc. pnts.   (vice-versa in brackets)  P3\_A: 21.2 (10.7)   P3\_L: 21.7 (11.4)   P2\_S: 18.0 (15.4)   P1prime\_Q: 19.5 (21.3)   P2prime\_Q: 11.2 (12.2) |  |
| **P3prime\_T**  14 in 199 sites   7.0 %    effects > 10 perc. pnts.   (vice-versa in brackets)  P1\_Q: 23.1 (29.4)   P1prime\_F: 10.3 (18.0)   P1prime\_M: 11.3 (26.3) |  |
